# Long-Term Treatment with Simvastatin Leads to Reduced Migration Capacity of Prostate Cancer Cells

**DOI:** 10.3390/biomedicines11010029

**Published:** 2022-12-22

**Authors:** Mona Kafka, Rebecca Gruber, Hannes Neuwirt, Michael Ladurner, Iris E. Eder

**Affiliations:** 1 Department of Urology, Medical University Innsbruck, Anichstraße 35, A-6020 Innsbruck, Austria; 2 Department of Internal Medicine IV Nephrology and Hypertension, Medical University Innsbruck, Anichstraße 35, A-6020 Innsbruck, Austria

**Keywords:** statins, prostate cancer, metastasis, cell migration, adhesion, cholesterol, long-term treatment

## Abstract

Statins have been shown to improve survival of metastatic prostate cancer (mPCa). Nevertheless, their therapeutic use is still under debate. In the present study, we investigated the short-term effects of three different statins (simvastatin, atorvastatin and rosuvastatin) in various PCa cell lines mimicking androgen-sensitive and -insensitive PCa. Moreover, we generated three new PCa cell lines (LNCaPsim, ABLsim, PC-3sim) that were cultured with simvastatin over several months. Our data showed that the three statins expressed highly diverse short-term effects, with the strongest growth-inhibitory effect from simvastatin in PC-3 cells and almost no effect from rosuvastatin in any of the cell lines. Long-term treatment with simvastatin resulted in a loss of response to statins in all three cell lines, which was associated with an upregulation of cholesterol and fatty acid pathways as revealed through RNA sequencing. Despite that, long-term treated cells exhibited diminished spheroid growth and significantly reduced migration capacity per se and to differentiated osteoclasts. These findings were strengthened by reduced expression of genes annotated to cell adhesion and migration after long-term simvastatin treatment. Notably, mPCa patients taking statins were found to have lower numbers of circulating tumor cells in their blood with reduced levels of PSA and alkaline phosphatase. Our data suggest that long-term usage of simvastatin hampers the metastatic potential of PCa cells and may therefore be a potential therapeutic drug for mPCa.

## 1. Introduction

With an incidence of >220/100,000 men, prostate cancer (PCa) represents one of the most common malignancies in developed countries [1]. Since the implementation of prevention screening programs, a diagnosis is often feasible in a locally restricted state with high cure rates. However, tumor biology varies from slow-growing forms to highly aggressive cancers with fast progression to metastatic states or even presentation as primary metastatic disease. Therefore, PCa is still a major cause of morbidity and mortality worldwide [1,2]. Based on the hormone dependency of PCa, androgen-deprivation therapy (ADT) accomplished by surgical or chemical castration is the gold standard therapy from the moment of metastatic disease. Since the implementation of several new hormonal agents (abiraterone acetate, enzalutamide, apalutamide, etc.) and chemotherapy in metastatic hormone-sensitive PCa (mHSPC), a combined therapy regime—whenever possible—represents the new state of the art. Unfortunately, a progression to the so-called stage of castration-resistant PCa (CRPC) occurs within approximately 18 to 36 months [3,4]. Within this stage of the disease, additional treatment options such as PARP (poly(ADP)-ribose polymerase)-inhibitors, Radium-223 or ^177^Lutetium-PSMA (prostate-specific membrane antigen) radionuclide therapy are approved [5].

Statins belong to the most prescribed drugs worldwide and successfully achieve a reduction of blood cholesterol levels by inhibition of the 3-hydroxy-3-methyl-glutaryl-coenzyme-A-reductase (HMGCR), a key enzyme of cholesterol synthesis. The following relative lack of cholesterol further leads to an increased expression of low-density-lipoprotein (LDL) receptors and consequently to an increased uptake of LDL with a subsequent further reduction of cholesterol levels [6]. Besides this effect, statins also exhibit anti-inflammatory, anti-oxidative and anti-proliferative effects, and were even shown to modulate the immune system in vitro [7]. Based on this so-called pleiotropic effect, statins are currently in the center of oncological research regarding a potential use in the treatment of diverse malignancies. Particularly referring to PCa, large epidemiological studies showed a decreased risk (up to 25–43%) to develop advanced PCa among statin users [8,9,10]. Moreover, there are various studies demonstrating that statin intake leads to a reduction of serum prostate specific antigen (PSA) levels and consequently to lower numbers of prostate biopsies and a PCa diagnosis [6,11,12]. Regarding metastatic disease, one recently published meta-analysis including 955 patients compared the clinical outcome of a treatment with novel antiandrogens with or without a combination with statins among patients with mCRPC. Although the type, dosage and duration of statin use was incompletely documented, there was a trend towards an improved overall survival (OS) among the statin group [13]. Another retrospective study showed that the use of statins in men with metabolic syndrome receiving ADT for the treatment of mHSPC prolonged the time to progression to CRPC [14]. On the contrary, there are also studies depicting that anti-cholesterolemic medication is associated with an increased risk for PCa and increased PCa aggressiveness [15,16]. A possible explanation for the conflicting data in the literature might be the broad and undifferentiated use of pharmacologically different anti-cholesterolemic drugs, which significantly differ in their pharmacological ways of action and may therefore exert different effects on tumor cells [17,18]. Furthermore, in clinical practice, statins are often combined with other medication such as acetylsalicylic acid (ASS) for the treatment of metabolic syndrome, and therefore a differentiated assessment of the effect may be limited. Additionally, no regular measurements of LDL blood levels are performed in urologic follow-up, so the interpretation of the actual effect of statins in PCa patients may be significantly confounded by patient compliance. Hence, it is still largely unclear whether statins may prohibit or in fact stimulate PCa development and/or progression and at which state of the disease their therapeutic use might be feasible.

In vitro studies showed that PCa cells are inhibited by short-term treatment with statins over a few days. These studies mostly included lovastatin and simvastatin [19,20]. Long-term effects of statins, however, were barely investigated, although statins represent a long-term and often life-long medication in clinical routine. In the present study, we therefore aimed to reveal not only short-term but also long-term effects of various statins on PCa cell growth using different cell lines, mimicking hormone-sensitive as well as castration-resistant mPCa. 

## 2. Materials and Methods

### 2.1. Cell Lines and Reagents

22Rv1, PC-3 and LNCaP cells were obtained from the American Type Culture Collection (ATCC, Rockville, MD, USA) and cultured in RPMI 1640 (PAN-Biotech, Aidenbach, Germany) with 10% fetal calf serum (FCS, Gibco, Grand Island, NY, USA), 1% GlutaMAX^TM^ (Gibco) and 1% penicillin and streptomycin (Lonza, Basel, Switzerland). The androgen-ablated subline LNCaPabl was previously established by long-term culture in androgen-ablated medium and maintained in RPMI 1640 with 10% charcoal-stripped (CS) FCS, 1% GlutaMAX^TM^ (Gibco), and 1% penicillin and streptomycin as described by Culig et al. [21]. All cell lines were cultivated at 37 °C in a humidified atmosphere with 5% CO_2_.

Long-term simvastatin-treated cells (LNCaPsim, ABLsim and PC-3sim) were established by culturing LNCaP, LNCaPabl and PC-3 parental cells with increasing (1–5 µM) concentrations of simvastatin over a period of 6 months. The final concentration of simvastatin was 5 µM. Control cells were maintained in medium with dimethyl sulfoxide (DMSO) in parallel. 

THP-1 human monocytes were obtained from Christian Ploner (Dept. of Plastic, Reconstructive and Aesthetic Surgery) and cultured in RPMI-1640 medium supplemented with 10% fetal bovine serum, 1% penicillin/streptomycin, and 1% GlutaMAX^TM^. Cells were routinely maintained at 37 °C in a humidified 5% CO_2_ atmosphere. To induce differentiation into osteoclasts, suspended cells were incubated over 2 days with 50 ng/mL phorbol 12-myristate 13-acetate (PMA) to obtain macrophages and then stimulated with 50 ng/mL receptor activator of nuclear factor k-B ligand (RANKL) and 25 ng/mL macrophage colony-stimulating factor (M-CSF) over 8 days.

Enzalutamide, atorvastatin, rosuvastatin, docetaxel, cabazitaxel (all from THP Medical Products, Vienna, Austria), apalutamide (Selleck Chemicals, Planegg, Germany), abiraterone acetate (Hycultec, Beutelsbach Germany), and simvastatin (Sigma/Merck, Darmstadt, Germany) were dissolved in DMSO (Sigma). 

### 2.2. Cell Viability and Spheroid Culture

Cell viability was determined via CellTiter 96^®^ Aqueous one solution cell proliferation assay (Promega). Briefly, 10 µL of reagent was added to 100 µL of cell culture medium and absorbance was measured at 490 nm on a Cytation™ 5 Cell Imaging Multi-Mode Reader (BioTek Instruments, Bad Friedrichshall, Germany). In each individual experiment, changes in cell viability were expressed as percentage of mock control. Three-dimensional spheroids were established as described previously [19]. Briefly, 8000 cells were seeded into each well of a 96-well ULC ultralow attachment plate (Costar, 7007, Sigma) and cultivated at 37 °C in a humidified atmosphere with 5% CO_2_. Spheroid size was determined with IncuCyte^®^ S3 LiveCell Analysis System.

### 2.3. Real Time Quantitative RT-PCR (qPCR)

Total RNA was isolated from cells with ExtractMe total RNA isolation Kit (Blirt, Gdansk, Poland) and quantified with a NanoDrop spectrophotometer (ND-2000c, Thermo Fisher Scientific, Vienna, Austria). RNA was transcribed into cDNA by reverse transcription using LunaScript RT SuperMix Kit (New England Biolabs, Frankfurt, Germany). The qPCR was performed with TaqMan™ expression assays (Thermo Fisher Scientific) for the quantification of *HMGCR* (Hs00168352_m1), *3-hydroxy-3-methylglutaryl-coenzyme A synthase 1* (*HMGCS1*, Hs00940429_m1) and *HMGCS2* (Hs00985427_m1), *hydroxymethylbilane synthase* (*HMBS*, Hs00609297_m1), *ATP citrate lyase* (*ACLY*, Hs00982738_m1), *fatty acid synthase* (*FASN*, Hs01005622_m1), *plasminogen activator*, *urokinase* (*PLAU*, Hs01547054_m1), *trombospondin-1* (*THBS1*, Hs00962908_m1), *fibronectin-1* (*FN1*, Hs01549976_m1), and *integrin subunit beta 4 (ITGB4*, Hs00236216_m1). The qPCR was carried out with ABI Prism 7500 Fast RT-PCR System (Thermo Fisher Scientific) cycler. Fold change in gene expression was determined using the mathematical model ratio 2^−ΔΔCT^. Values of genes of interest (GOI) were determined relative to *HMBS*. Fold change expression was calculated relative to the mock control for each individual experiment.

### 2.4. RNA Sequencing and Data Analysis 

Following long-term treatment with 5 µM simvastatin, cell lines were tested free of mycoplasma. Cell line identity was checked with the use of short tandem repeat analysis at the Institute of Forensic Medicine (Prof. W. Parson, Innsbruck, Austria). RNA was isolated with the ExtractMe total RNA isolation Kit (Blirt, Gdansk, Poland). RIN (RNA integrity number) was determined with Agilent 2100 bioanalyzer. For RNA sequencing (RNAseq), RNA samples had to meet the following criteria: at least 2 µg RNA and a RIN >8. Library preparation and RNAseq (Illumina NovaSeq 2 × 150 sequencing, polyA selection, 350M read pairs) as well as raw data evaluation was conducted by GENEWIZ GmbH (Leibzig, Germany). Differentially expressed genes (DEGs) were identified between control (DMSO) and simvastatin (sim) treated cells from three biological replicates when the corrected *p*-value was <0.05 and the fold change >2. Gene ontology pathway analysis was performed using PANTHER Overrepresentation Test (Released 24 February 2021) assessed on 3 August 2021 (http://pantherdb.org/tools/) to receive a list of pathways significantly altered in long-term treated cells. Potential targets were selected on whether they were significantly up- or downregulated (at least 2-fold) in simvastatin versus control cells. Datasets are available under digital supplementary data (Additional file S1. Array Data co vs. sim.xlsx, Additional file S1).

### 2.5. Caspase 3/7 Assay

Caspase 3/7 activities were determined with a Caspase-Glo^®^ 3/7 Assay (Promega, Madison, WI, USA). Briefly, cells were plated in a 96-well plate. Following treatment, 100 µL of Caspase Glo^®^ Reagent was added per well and luminescence was measured after 1 h on a Cytation™ 5 Cell Imaging Multi-Mode Reader (BioTek Instruments). Caspase 3/7 activity was expressed as percentage of mock control that was set 100%.

### 2.6. Cholesterol/Cholesterol Ester-Glo™ Assay

To determine intracellular cholesterol levels, a Cholesterol/Cholesterol Ester-Glo^TM^ Assay (Promega) was performed according to the manufacturer’s instructions. In brief, 20,000 cells were seeded per well in a 96-well plate in RPMI + 10% CS FCS. After 72 h of treatment, medium was removed and the cells were lysed with 50 µL Cholesterol Lysis Solution per well for 30 min at 37 °C. The cell lysis solution was then transferred into another 96-well plate (Corning 3610, white, clear bottom) and mixed with Cholesterol Detection Reagent in the presence of esterase. After incubation for 1 h at room temperature, chemoluminescence was measured on a Cytation™ 5 Cell Imaging Multi-Mode Reader (BioTek Instruments). Total cholesterol levels were normalized to protein content that was determined by Pierce^TM^ BCA protein assay (Thermo Fisher Scientific) and expressed as µM cholesterol.

### 2.7. Migration Assay

Cells (30,000 cells/well) were seeded into Corning FluoroBlok inserts (8.0 µm pore size, Szabo Scandic) in 500 µL serum-free RPMI per well and placed into 24-well companion plates (Thermo Fisher Scientific). The lower chamber was filled with 650 µL RPMI + 10% FCS. To estimate the amount of seeded cells and to determine the percentage of migrated cells, cells were seeded in parallel in one well without insert. To investigate migration of PC-3 cells towards differentiated osteoclasts, THP-1 cells (30,000 cells/well) were first seeded into a 24-well companion plate and differentiated over 8 days in the presence of RANKL (50 ng/mL) and M-CSF (25 ng/mL). Then, PC-3 cells were seeded into the inserts as described above. After 72 h, the medium from both chambers was aspirated. Migrated cells were stained with a 2 µM calcein solution in HBSS + 0.1% bovine serum albumin (BSA) in the lower chamber and incubated for 1 h at 37 °C. Then, the calcein solution was replaced against 500 µL HBSS + 0.1% BSA. Fluorescence was measured at a wavelength of 485/528 nm on a Cytation™ 5 Cell Imaging Multi-Mode Reader (BioTek Instruments). Representative images were taken on a JuLI Smart Fluorescence Cell Imager microscope (Digital Bio).

### 2.8. Counting of Patient-Derived Circulating Tumor Cells (CTCs)

CTCs were isolated and counted as described previously [22]. Briefly, blood was collected from mPCa patients into TransFix tubes (Cytomark, Buckingham, UK) and enumerated with a Parsortix^TM^ microfluidic system. Afterwards, CTCs were identified and counted upon positive prostate-specific membrane antigen (PSMA) and pan-cytokeratin and negative CD45 staining using a Zeiss Axio Observer A1 (Zeiss Microscope, Jena, Germany). The study was carried out in accordance with ethical approval from the Medical University Innsbruck (Approval Number 2014-0021, UN4837). Written informed consent was obtained from all participants prior to blood draw. Patients with mPCa were subdivided into a group of statin users (n = 19) and another group that did not take any statin (n = 41). In the group of statin users, 13 patients took simvastatin, 1 patient atorvastatin, 2 patients rosuvastatin, 2 patients pravastatin and 1 patient received fluvastatin.

### 2.9. Statistics

Statistical calculations were performed with IBM SPSS Statistics 27 software and determined by Mann–Whitney-U test (nonparametric test with 2 independent samples). Data were expressed as means with standard error of mean (SEM) unless otherwise stated; * *p* < 0.05 was considered statistically significant; ** *p* < 0.01; *** *p* < 0.001.

## 3. Results

### 3.1. Differential Effects of 3 Different Hydrophilic and Lipophilic Statins with or without Enzalutamide on Prostate Cancer Cells

We first investigated the short-term effects of three different statins on various PCa cell lines, representing different stages of the disease: androgen-sensitive LNCaP cells, androgen-insensitive PC-3 cells and two cell lines mimicking castration resistance (LNCaPabl, 22Rv1). We selected three widely prescribed statins (simvastatin, atorvastatin and rosuvastatin) because of their different pharmaceutical characteristics as reviewed by Althanoon [18]. Simvastatin and atorvastatin are lipophilic pro drugs, which are metabolized through cytochrome P450 CYP4A5, whereas rosuvastatin is hydrophilic and does not need an additional activation step. In addition, atorvastatin and rosuvastatin both use the organic anion transporting polypeptide OATP1P1 for cellular uptake [18]. 

Cell viability was determined after treatment of cells with rising concentrations of each statin ranging from 0.1 to 5 µM over 3 days. We tested these concentrations as they were commonly used in preclinical trials on statins in PCa [23,24]. Of note, a previous pharmacological study showed that even high doses of lovastatin that are required to reach plasma bioactivity levels in this µM range are well-tolerated [25]. 

**Figure 1:**
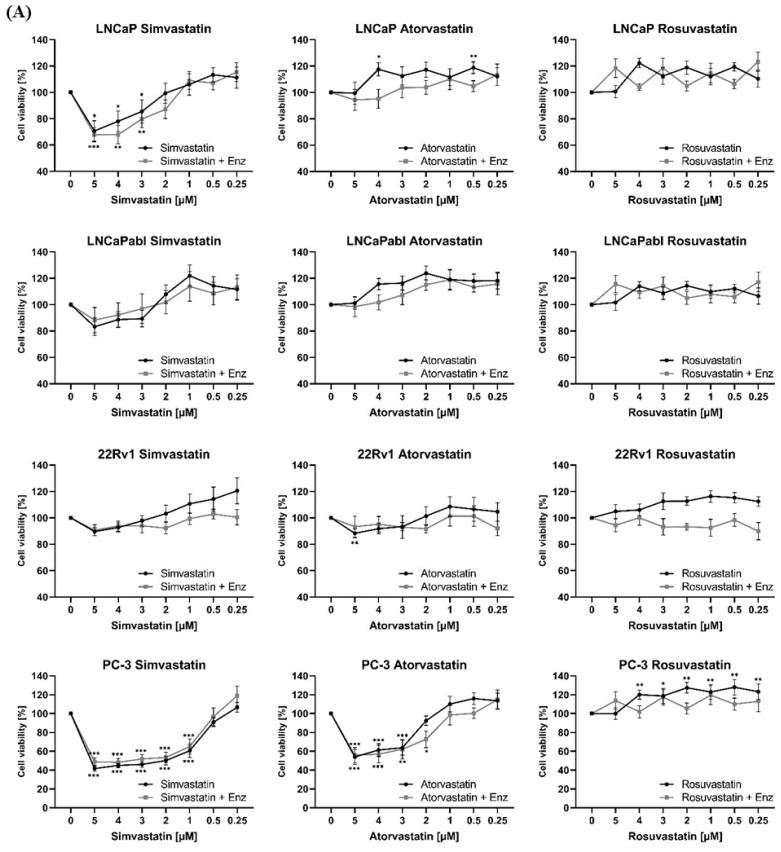

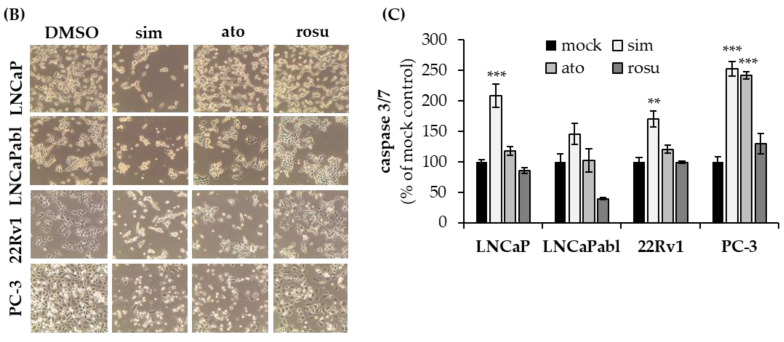
Growth-inhibitory effects of statins on different PCa cell lines. (**A**) Four different PCa cell lines (LNCaP, LNCaPabl, 22Rv1, PC-3) were treated with increasing concentrations of simvastatin (sim), atorvastatin (ato), and rosuvastatin (rosu) in the absence or presence of 5 µM enzalutamide (enza) over 72 h. Cell viability was assessed with a colorimetric CellTiter 96^®^ Aqueous one solution cell proliferation assay and expressed as percentage of mock control (DMSO) that was set 100%. (**B**) Representative images were taken after 72 h of treatment with 5 µM of the indicated statin (100× magnification). (**C**) Induction of apoptosis was assessed after treatment of cells with 5 µM of each statin through a caspase 3/7 assay. Caspase 3/7 activity was expressed as percentage of mock control that was set 100%. Data are represented as mean ± SEM. (** *p* < 0.01, *** *p* < 0.001).

Simvastatin demonstrated the strongest growth inhibitory effect of all three statins, followed by atorvastatin and rosuvastatin, which in fact did not express any significant inhibition on the investigated cell lines (Figure 1A). At a concentration of 1 µM, simvastatin accomplished a >50% reduction of cell growth representing the IC50, whereas atorvastatin reached the IC50 mark at a concentration of 3 µM in the same cell lines. Responsiveness to the three statins also strongly differed among the cell lines. Simvastatin and atorvastatin had the strongest growth-inhibitory effect in androgen-insensitive PC-3 cells, followed by LNCaP cells, which mimic hormone-sensitive PCa. The two castration-resistant cell lines, LNCaPabl and 22Rv1, on the other hand, were clearly less sensitive to statin treatment. Representative images of each cell line after treatment with the most effective dose of 5 µM are shown in Figure 1B. 

Corresponding with the effect obtained on cell viability, simvastatin significantly induced apoptosis in LNCaP and PC-3 cells and to a much lesser extend in LNCaPabl and 22Rv1 cells (Figure 1C). Atorvastatin, by contrast, only induced apoptosis in PC-3 cells whereas rosuvastatin did not induce apoptosis in any of the cell lines (Figure 1A,B). 

In combination with 5 µM of the anti-androgen enzalutamide, there was a weak increase in growth inhibition of androgen-sensitive LNCaP cells compared to statins alone, particularly at lower concentrations between 2 and 4 µM (Figure 1A), whereas there was no apparent additive effect of statins and enzalutamide in PC-3 cells. In castration-resistant LNCaPabl cells, enzalutamide combined with atorvastatin resulted in the most prominent growth inhibition. Notably, simvastatin and also rosuvastatin were able to increase the effect of enzalutamide in 22Rv1 cells. These data suggest that statins may in fact be able to enhance the tumor growth inhibitory effect of enzalutamide, however, depending on the type of statin and the tumor cell line.

### 3.2. Differential Effects of Statins on the Expression of HMGCR 

**Figure 2:**
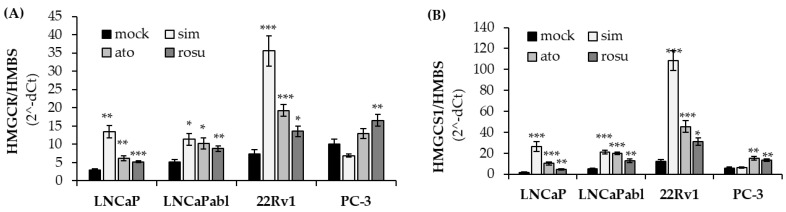
Expression of *HMGCR* (**A**) and *HMGCS1* (**B**) after treatment with statins (5 µM) was determined via real-time qPCR and normalized to the housekeeping gene *hydroxybilane synthase* (*HMBS*). Data are represented as mean ± SEM. (* *p* < 0.05, ** *p* < 0.01, *** *p* < 0.001).

One of the possible explanations for the differential effects of statins observed in PCa cells is the transcriptional upregulation of enzymes of the mevalonate pathway by sterol regulatory element binding protein 2 (SREBP2), a feedback mechanism that is activated by statin treatment. Previous studies have demonstrated that AR-negative PC-3 cells lack SREBP2 expression and are therefore highly responsive to statins [26]. Therefore, we next investigated changes in *HMGCR* expression after treatment of our 4 PCa cell lines with the three different statins through qPCR. As depicted in Figure 2A, simvastatin treatment induced an upregulation of *HMGCR* in AR-positive LNCaP, LNCaPabl and 22Rv1 cells but not in AR-negative PC-3 cells, confirming previously published results [26]. Similarly, *HMGCS1* was significantly upregulated by simvastatin in LNCaP, LNCaPabl and 22Rv1 cells but did not affect the expression in PC-3 cells (Figure 2B). Treatment with atorvastatin and rosuvastatin, by contrast, increased the expression of *HMGCR* and *HMGCS1* in all 4 cell lines, including PC-3 cells, indicating that a lack of response to the different statins cannot solely be explained by the SREBP2 mediated feedback mechanism.

### 3.3. Long-Term Effects of Simvastatin 

**Figure 3:**
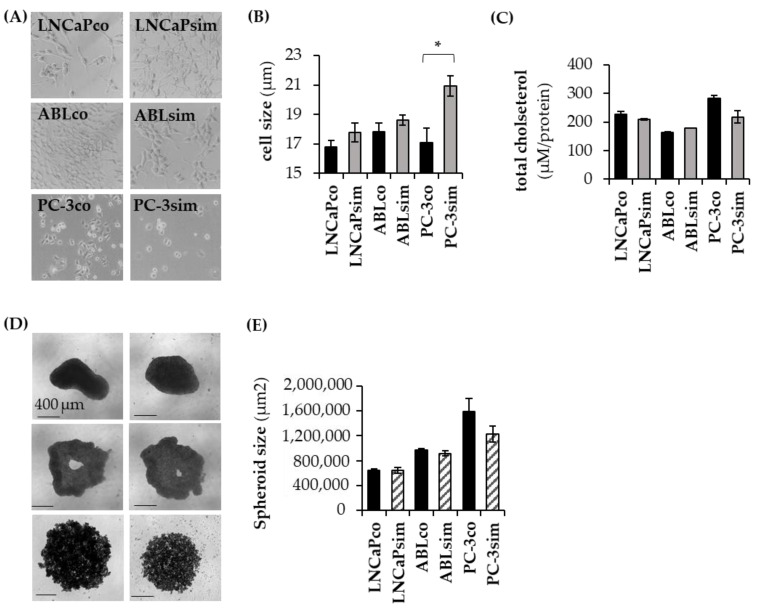
Morphological changes following long-term simvastatin treatment. (**A**) Representative images were taken from each cell line. Magnification 100×. (**B**) Cell size was determined with a Casy cell counter and analyzer. (**C**) Cholesterol levels were determined with a bioluminescent assay as described under material and methods. Total cholesterol levels were normalized to protein content that was determined via BCA assay. (**D,E**) Cells were seeded into Corning 7007 96-well plates to form 3-dimensional spheroids. After 8 days of culture, spheroid size was determined with an image-based brightfield IncuCyte^®^ S3 LiveCell Analysis System. Data are represented as mean ± SEM. (* *p* < 0.05).

Since simvastatin exhibited the strongest anti-proliferative effect on PCa cells in our short-term experiments, we used this statin to study the effects of long-term treatment on PCa cell growth. For that reason, LNCaP, LNCaPabl and PC-3 cells were cultivated in a medium containing simvastatin over a period of 6 months. Due to the strong growth-inhibitory effect of simvastatin, cells were adopted to increasing concentrations of the drug step by step up to a final concentration of 5 µM. Corresponding with the observed short-term effects, PC-3 cells exhibited a higher drug sensitivity and therefore required a slower rise in simvastatin concentration compared to the other cell lines. The newly established cell lines were designated LNCaPsim, ABLsim, and PC-3sim. Overall, we recognized a change in cell morphology with a tendency to a greater cell size in long-term simvastatin-treated cells as compared to the parental cells. However, this difference was only statistically significant in PC-3sim cells (Figure 3A,B). Of note, this increase in cell size was not associated with an increase in the cellular cholesterol content (Figure 3C). Moreover, 3D spheroid size of PC-3sim cells was reduced compared to their parental controls (Figure 3D). 

**Figure 4:**
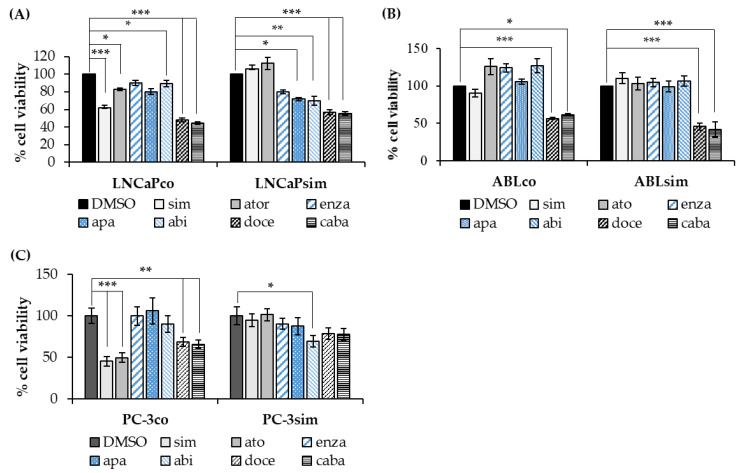
Effects of statins, antiandrogens and chemotherapeutics on long-term simvastatin-treated cell lines. LNCaP (**A**), ABL (**B**), and PC-3 (**C**) cells were treated with 5 µM simvastatin (sim), 5 µM atorvastatin (ato), 5 µM enzalutamide (enza), 5 µM apalutamide (apa), 5 µM abiraterone acetate (abi), 12 nM docetaxel (doce) and 12 nM cabazitaxel (caba) over 72 h and viability was measured with a colorimetric cell viability assay. Cell viability was expressed as percentage of mock control. Data are represented as mean ± SEM. (* *p* < 0.05, ** *p* < 0.01, *** *p* < 0.001).

As shown in Figure 4, the long-term simvastatin-treated cell lines were resistant to the growth-inhibitory effect of simvastatin and atorvastatin compared to their respective parental cells (Figure 4). Of note, however, LNCaPsim cells seemed to become more sensitive to the anti-androgens enzalutamide, apalutamide and abiraterone (Figure 4A). ABLsim, on the other hand, were not inhibited by the anti-androgens, though also not stimulated, a phenomenon that was observed in ABLco cells (Figure 4B). All three long-term simvastatin-treated cell lines still responded to treatment with the chemotherapeutic drugs docetaxel and cabazitaxel, although PC-3sim cells seemed to be less sensitive compared to PC-3co cells (Figure 4C). In castration-resistant ABLsim, by contrast, the effect of docetaxel and cabazitaxel was even stronger than in ABLco cells. 

**Figure 5:**
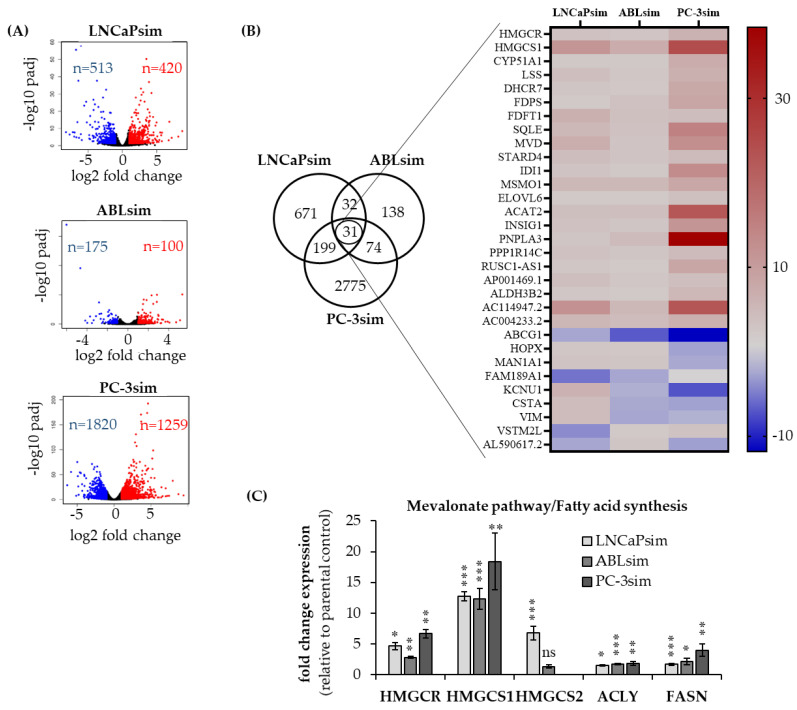
Identification of differentially expressed genes following long-term simvastatin treatment. (**A**) Volcano plot of differentially expressed genes (DEGs) in control and simvastatin (sim)-treated cells. Red points represent upregulated, blue points downregulated genes. Genes without any significant difference are in black. (**B**) Heat map illustrating 31 DEGs in all 3 cell lines following long-term simvastatin treatment. (**C**) Fold change in gene expression in long-term simvastatin-treated cell lines compared to their parental control cells determined by real-time qPCR in 3 different passage numbers of simvastatin-treated cells. Data are represented as mean ± SEM. (* *p* < 0.05, ** *p* < 0.01, *** *p* < 0.001, ns, not significant).

To further characterize our newly established long-term simvastatin-treated PCa cell lines and to gain more insight into possible changes on gene expression by long-term statin treatment, we performed an RNAseq of long-term treated and their corresponding parental cells. We found a substantial number of differentially expressed genes (DEGs) after long-term simvastatin treatment in all three cell lines as summarized in Figure 5A. The highest number of gene expression changes was observed in PC-3sim cells with 1259 up- and 1820 downregulated genes. In LNCaPsim, the number of DEGs was lower than in PC-3sim with 420 up- and 513 downregulated genes. In ABLsim cells, only 100 genes were up- and 175 genes were downregulated, thereby showing the weakest effect of simvastatin long-term exposure on gene expression. 

Out of all DEGs, we identified 31 genes, which were similarly altered in all three long-term simvastatin-treated cell lines compared to their parental controls. We noticed that most of these genes (22/31) were significantly upregulated by simvastatin treatment and mostly were related to cholesterol, fatty acid and sterol biosynthesis pathways, including *HMGCR*, *HMGCS1*, *squalene monooxygenase* (*SQLE*), *insulin induced gene 1* (*INSIG1*) and *acetyl-CoA acetyltransferase 2* (*ACAT2*) (Figure 5B). Additionally, *the cholesterol efflux transporter ABCG1* was downregulated in all three cell lines. 

To further validate the data gained by RNAseq, a qPCR with the focus on the expression of genes of the cholesterol pathway was performed in the long-term simvastatin-treated cell lines and compared with that of the parental controls. As depicted in Figure 5C, we confirmed a significant upregulation *of HMGCS1*, *HMGCR*, *ACLY*, and *fatty acid synthase* (*FASN*) in LNCaPsim, ABLsim, and PC-3sim compared to the controls. These data suggest that long-term simvastatin treatment upregulates the expression of genes related to cholesterol, fatty acid and steroid biosynthesis.

**Figure 6:**
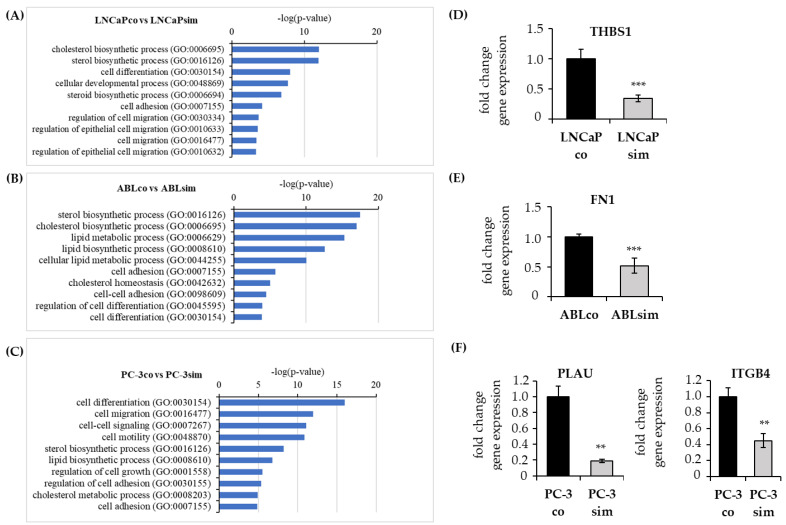
Changes in the expression of genes related to cell migration and cell adhesion. (**A**–**C**) Top 10 significantly regulated pathways as identified through GO pathway analysis. Expression of (**D**) *thrombospondin-1* (*THBS1*), (**E**) *fibronectin* (*FN-1*), (**F**) *urokinase plasminogene activator* (*PLAU*), *integrin subunit beta 4* (*ITGB4*), and *urokinase plasminogene activator* (*PLAU*) annotated to “cell adhesion” and “cell migration” was determined in long-term simvastatin-treated cells through real-time qPCR and compared to parental control cells. Data are represented as mean ± SEM. (** *p* < 0.01, *** *p* < 0.001).

Further gene ontology (GO) pathway analysis interestingly revealed that besides cholesterol biosynthesis, cell adhesion and migration were among the most significantly altered pathways in long-term simvastatin-treated cells (Figure 6A–C). Among those genes annotated to cell adhesion and migration, a significant number were significantly downregulated in simvastatin-treated compared to the control cells (Table 1, Table 2 and Table 3).

We selected the most significantly downregulated genes in each cell line and confirmed their expression by real-time qPCR (Figure 6D–G). As revealed by RNAseq, *thrombospondin-1* (*THBS1*) was significantly downregulated in LNCaPsim compared to LNCaPco, whereas *fibronectin-1* (*FN-1*) was significantly downregulated in ABLsim compared to ABLco. In PC-3sim, *urokinase plasminogen activator* (*PLAU*) and *integrin subunit beta 4* (*ITGB4*) were significantly lower expressed compared to PC-3co cells. Furthermore, downregulation of urokinase plasminogen activator (uPA) was also shown on protein-level in our long-term treated PC-3sim cells, as determined by ELISA (Supplementary Figure S1). Notably, uPA levels could not be determined in LNCaP and ABL cells because they were under the detection limit of the assay. We further noticed that—among the three long-term simvastatin-treated cell lines—the number of downregulated genes related to cell adhesion and migration was the lowest in ABLsim.

**Figure 7:**
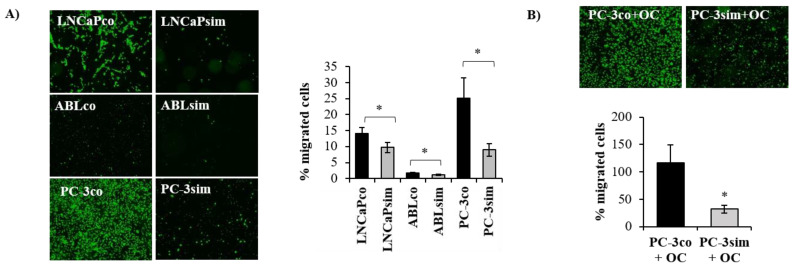
Long-term simvastatin treatment reduced the migration capacity of PCa cells per se and towards osteoclasts. (**A**) Cells were seeded into 24-well fluoroblok transwell inserts as described under material and methods in serum-starved culture medium and allowed to migrate through the membrane towards medium containing 10% fetal calf serum for 72 h. Following staining with calcein, representative images were taken with a JuLi live cell imager (4× magnification). Fluorescence of migrated cells was quantified with a Cytation™ 5 Cell Imaging Multi-Mode Reader and expressed as percentage of migrated cells. (**B**) PC-3co and PC-3sim cells were seeded into 24-well plate Fluoroblok transwell inserts in serum-starved medium and allowed to migrate towards the lower chamber where differentiated osteoclasts (OC) were cultured over 8 days in the presence of PMA (50 ng/mL), RANKL (50 ng/mL), and M-CSF (25 ng/mL). Data are represented as mean ± SEM. (* *p* < 0.05).

Overall, these data suggest that despite the acquired insensitivity of long-term simvastatin-treated cells to statins and the significant increase in the expression of cholesterol pathway genes, these cells are presumably affected in their cell adhesion and migration abilities. Based on this assumption, we next investigated the migration capacity of simvastatin-treated cell lines using a FluoroBlok migration assay. As shown in Figure 7A, all three cell lines exhibited a significantly reduced migration capacity compared to their parental controls. The effect was again most pronounced in PC-3sim cells. In general, PC-3co cells had a higher percentage of migrating cells than LNCaPco and ABLco cells. ABLsim cells also had a reduced migration capacity compared to ABLco cells; however, these cells migrated weakly compared to LNCaP and PC-3 cells. Since PC-3 cells are derived from a bone metastasis, we next investigated their migration towards differentiated osteoclasts (OC). To this end, human THP-1 monocytes were cultured with PMA, RANKL, and M-CSF over 9 days in the lower chamber as described under material and methods. Then, PC-3co and PC-3sim cells were seeded into fluoroblok transwell inserts, which were added to the wells with osteoclasts of the companion plate. After another 72 h, the migration of PC-3co and PC-3sim was determined by calcein staining of the insert. As shown in Figure 7B, the percentage of migrated cells towards osteoclasts was significantly lower in PC-3sim cells compared to the parental PC-3co cells (Figure 7B), indicating that long-term simvastatin treatment impairs migration of PCa cells to the bone.

### 3.4. Reduced Number of Circulating Tumor Cells and Reduced Alkaline Phosphatase in mPCa Patients Taking Statins

**Figure 8:**
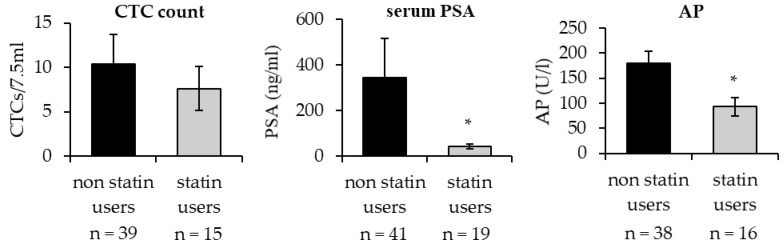
Reduced circulating tumor cell (CTC) count in mPCa patients taking statins. Blood from patients with mPCa (n = 60) was analyzed for CTC count, serum prostate specific antigen (PSA) and alkaline phosphatase (AP) and grouped into statin users and non-statin users (* *p* < 0.05).

Based on these findings, we assumed that statins could impair tumor progression in patients with mPCa. To further strengthen our hypothesis, we next analyzed the number of circulating tumor cells (CTCs) in patients with mPCa taking statins and compared them with non-statin users. To this end, we looked at the data from a previous study that was recently published by our group [22]. As shown in Figure 8, the number of CTCs in the peripheral blood was reduced in statin users compared to non-users, though the difference between the two groups was not statistically significant. Importantly, alkaline phosphatase (AP), a marker that indicates the extent of bone metastasis, as well as PSA were significantly lower in the statin user group compared to non-statin users. 

## 4. Discussion

Statins express a variety of different effects and are therefore currently in the focus of research in various oncologic areas of expertise [27,28,29]. A recently published review summarizing findings of prospective and registry-based studies on statins and PCa concluded that there is an overall lower risk of advanced and fatal PCa among statin users and also a better outcome among PCa patients with statin therapy. The anti-cancer effect of statins, however, may largely depend on tumor-specific molecular characteristics, making it difficult to estimate which patients would in fact benefit from statin treatment [30]. Hence, the effect of statins on the development and progression of PCa is still unclear. Within our study, we found significant differences among three frequently prescribed statins (simvastatin, atorvastatin and rosuvastatin) with regard to their growth-inhibitory effects in PCa cells. Overall, simvastatin was the most efficient anti-proliferative drug, followed by atorvastatin, whereas rosuvastatin did not exhibit any significant effects in our study. Of note, we also found varying responses in the different cell lines mimicking androgen-sensitive as well as androgen insensitive stages of the disease, indicating that the response of PCa cells to statin treatment might be very heterogeneous in general and even strongly dependent on the type of statin used. Moreover, a combination of statins with the antiandrogen enzalutamide did not significantly potentiate the anti-proliferative effect of statins alone when used only over 3 days and again was dependent on the type of statin and the cell line used. This finding might have an important consequence for the validation of clinical data where patients were categorized into statin users and non-statin users without any further differentiation among the different statin drugs [6,8,9,10,11,13,14,15,20]. 

As statins are a long-term and often even a life-long medication in patients with hypercholesterolemia or a cardiovascular risk profile, we aimed at generating a suitable model to study long-term statin treatment on PCa cells. With our newly established long-term simvastatin-treated PCa cell lines, LNCaPsim, ABLsim and PC-3sim, we present a suitable cell culture model to study long-term effects of statin medication on PCa cells. Our results demonstrate that long-term treatment with simvastatin renders PCa cells unresponsive to statins such as simvastatin and atorvastatin. Importantly, however, LNCaPsim cells were more sensitive to anti-androgens, including enzalutamide, apalutamide and abiraterone, compared to parental LNCaPco cells, indicating that simvastatin could be used to sensitize PCa cells to antiandrogen therapy. Suitable to our findings, Harshman et al. recently showed a significant prolonged time to progression (27.5 months vs. 17.4 months) in patients with hormone-sensitive PCa who had a co-medication with a statin at the start of ADT as compared to non-statin users [31]. A reduced risk of progression and even death of PCa in statin users was also demonstrated in other retrospective cohort studies [32,33]. Of note, long-term treatment with simvastatin did not alter the response to chemotherapeutics in our study. 

A molecular characterization of long-term simvastatin-treated cells through RNA sequencing revealed a broad variety of differentially expressed genes after long-term simvastatin treatment. A significant number of upregulated genes was annotated to fatty acid, cholesterol and steroid biosynthesis, a phenomenon that can also be observed by short-term statin treatment of PCa cells. The qPCR results strengthened these findings, showing a significant upregulation of *HMGCR*, *HMGCS1/2*, *ACLY* and *FASN*. This statin-mediated upregulation of cholesterol synthesis genes has been described previously in the literature. In fact, it was shown that statin treatment induces an upregulation of cholesterol genes as a negative feedback loop, which is regulated through steroid regulatory element binding protein 2 (SREBP2) in androgen receptor positive PCa cell lines, rendering them insensitive to statins. Androgen receptor negative PC-3 cells, on the other hand, are highly sensitive to statins because they are missing this negative feedback loop [34]. Importantly, our data revealed that cholesterol genes were upregulated in all three cell lines, including PC-3sim. In addition, whereas simvastatin did not affect the expression of *HMGCR* and *HMGCS2* as described in the literature, we could still see a significant upregulation of these genes by treatment with atorvastatin and rosuvastatin, indicating there may be another pathway besides SREPB2 that is responsible for the differential anti-proliferative effects in PCa cells. 

Altogether, long-term simvastatin treatment resulted in loss of response to statins and an upregulation of cholesterol and fatty acid synthesis, which let us assume that long-term statin medication would render the tumor cells more aggressive. By contrast, we observed that long-term simvastatin PCa cells did not form larger spheroids in 3-dimensional culture compared to parental control. PC-3sim spheroids were even smaller than those of PC-3co. Furthermore, gene expression analysis revealed that cell adhesion and cell migration were among the most significantly altered pathways in long-term treated PCa cells. In fact, a number of genes that are important for cell migration and cell adhesion, including *thrombospondin-1*, *fibronectin-1* and *urokinase plasminogen activator*, were significantly downregulated in long-term simvastatin-treated cells. Migration assays further confirmed that long-term treatment with simvastatin significantly reduced the migration capacity of PCa cells. This effect was again most pronounced in PC-3sim cells but also significant in androgen-sensitive LNCaP and castration-resistant ABL cells. Importantly, PC-3sim cells, which are derived from a bone metastasis, also exhibited a significantly reduced migration ability towards differentiated osteoclasts in an in vitro co-culture experiment, indicating that statins might reduce migration of PCa cells to the bone. These results were strengthened by the finding that alkaline phosphatase and PSA were significantly lower in mPCa patients taking statins compared to non-statin users. Moreover, statin users showed a trend towards lower CTC counts, suggesting that statins may in fact impair PCa metastasis to the bone. Recent literature described a reduced migration of PCa cells after treatment with atorvastatin, possibly through inhibition of the epithelia–mesenchymal transformation (EMT) and the expression of matrix metalloproteinase (MMP) expression as recently published by Zhu et al. [35]. 

## 5. Conclusions

In this study, we were able to show that long-term simvastatin treatment impairs the migration capacity of PCa cells. In vitro data were supported by clinical findings showing that mPCa patients taking statins have reduced CTC counts and lowered alkaline phosphatase and PSA levels. Moreover, due to the strong growth-inhibitory effect of statins in particular in bone-metastasis-derived PC-3 cells and impaired migration of long-term PC-3sim cells towards osteoclasts, we suggest that treatment of mPCa patients with statins in combination with actual therapy regimens might be beneficial for the patients and help in reducing metastatic tumor progression. Notably, our findings also demonstrate the large diversity among different statin drugs and PCa cell lines with respect to tumor growth inhibition, suggesting that only well-designed clinical trials will be able to find out whether statins might in fact be valuable therapeutics for PCa patients. Another important issue that needs to be considered with respect to a therapeutic effect of statins on PCa is the right dosage, since concentrations that are effective in vitro might not be reached in the plasma of patients with doses that are commonly used for cardiovascular diseases. There is a current phase-three clinical trial of the Prostate Cancer Consortium in Europe (PEACE) with one study arm investigating standard of care therapy combined with atorvastatin among patients with early metastasized CRPC (PEACE-4 trial) as stated on clinicaltrial.gov (assessed on 24 November 2022) [36]. Furthermore, there is another randomized placebo arm controlled clinical trial recruiting patients with hormone-sensitive PCa starting with ADT in combination with 80 mg atorvastatin daily [37]. Hopefully, results from these studies may affect further treatment regimes in the field of prostate cancer in the future.

## Figures and Tables

**Table 1:** List of significantly downregulated genes (fold change < 0.5, n = 3) annotated to cell adhesion and cell migration in LNCaPsim compared to LNCaPco cells.

	LNCaPco	LNCaPsim	Fold Change	*p*-Value
THBS1	23,397.3	7203.9	0.307	3.52 × 10^−10^
SEMA6D	3960.1	60.4	0.015	6.13 × 10^−63^
IGF1R	3107.4	1336.5	0.430	0.00048
NLGN1	1373.4	276.8	0.202	1.37 × 10^−9^
TMSB4X	1050.6	13.2	0.011	5.23 × 10^−42^
GPC6	908.1	230.8	0.254	4.65 × 10^−9^
PTK7	881.8	267.2	0.304	7.64 × 10^−16^
COL1A1	752.0	284.8	0.378	1.62 × 10^−9^
DOCK10	701.3	6.9	0.009	2.46 × 10^−7^
AMIGO1	477.9	221.2	0.463	5.45 × 10^−5^
SDK1	377.5	62.5	0.165	2.58 × 10^−13^
PCDHB5	254.1	74.6	0.293	9.55 × 10^−8^
UNC5C	248.9	98.2	0.392	3.30 × 10^−5^
CDH3	174.7	3.7	0.021	6.72 × 10^−27^
PCDHB2	121.8	34.4	0.283	4.75 × 10^−7^
PLXND1	79.2	30.9	0.390	4.99 × 10^−5^
NECTIN3	47.8	13.2	0.276	0.00070
NTN1	39.9	8.9	0.220	0.00225

**Table 2:** List of significantly downregulated genes (fold change < 0.5, n = 3) in ABLsim compared to ABLco cells.

	ABLco	ABLsim	Fold Change	*p*-Value
FN1	1756.6	589.2	0.336	6.54 × 10^−8^
COL6A2	300.1	109.5	0.358	0.00013
CDH15	102.3	11.7	0.482	0.00018
PCDH15	102.3	11.7	0.115	6.78 × 10^−17^
CNTN3	53.9	21.4	0.398	0.00064

**Table 3:** List of significantly downregulated genes (fold change < 0.5, n = 3) in PC-3sim compared to PC-3co cells.

	PC-3co	PC-3sim	Fold Change	*p*-Value
PLAU	390,694.9	80,503.1	0.206	5.81 × 10^−70^
ITGB4	144,623.7	69,692.8	0.481	4.80 × 10^−11^
ITGA3	61,337.3	27,100.7	0.441	1.90 × 10^−13^
LAMC2	20,784.5	8777.1	0.422	1.72 × 10^−16^
FUT8	16,498.0	7383.9	0.447	3.60 × 10^−12^
CDC42BPA	12,522.0	6012.6	0.480	7.15 × 10^−14^
SEMA4B	10,651.1	4542.9	0.426	1.10 × 10^−9^
AJUBA	10,409.0	4454.5	0.427	7.52 × 10^−26^
CDH11	9388.1	3316.2	0.353	4.72 × 10^−27^
FERMT2	8542.0	3858.6	0.451	1.41 × 10^−21^
CEACAM6	6315.0	1518.4	0.240	5.39 × 10^−5^
LAMA4	5233.2	2326.1	0.444	2.67 × 10^−10^
SLIT2	4432.6	1960.7	0.442	1.79 × 10^−11^
SHH	3186.3	986.6	0.309	0.00065
STC1	3030.1	1108.3	0.365	1.31 × 10^−18^
VEGFC	2695.9	1035.0	0.383	2.29 × 10^−27^
FYN	2367.5	987.2	0.416	2.44 × 10^−12^
CDH7	2226.0	399.4	0.179	2.44 × 10^−34^
DLC1	1815.7	383.8	0.211	3.33 × 10^−18^
KRT16	1190.2	307.0	0.257	6.81 × 10^−17^
COL17A1	1169.4	385.1	0.329	2.46 × 10^−27^
FAM110C	1147.5	244.4	0.212	3.33 × 10^−29^
CDH13	1110.2	112.6	0.101	1.16 × 10^−27^
KIRREL3	986.6	363.9	0.368	2.21 × 10^−10^
ECM2	924.7	232.2	0.251	6.38 × 10^−14^
HBEGF	892.2	411.4	0.460	0.00018
PDGFRA	827.0	294.4	0.356	2.90 × 10^−12^
KDR	667.3	197.4	0.295	1.53 × 10^−9^
PCDH9	520.0	180.4	0.347	1.34 × 10^−9^
FAT4	467.1	148.7	0.317	1.87 × 10^−11^
MMP10	370.2	115.7	0.312	3.64 × 10^−7^
ITGA8	347.7	74.2	0.213	0.00261
PSTPIP2	290.0	9.7	0.033	3.86 × 10^−50^
PRSS2	271.7	20.2	0.331	1.62 × 10^−26^
CADM2	264.5	62.2	0.235	1.64 × 10^−20^
SEMA6D	235.8	94.1	0.400	3.15 × 10^−10^
EPHA3	222.4	55.6	0.250	1.90 × 10^−11^
TNFAIP6	157.7	36.1	0.227	3.66 × 10^−8^
TNFSF18	148.9	13.7	0.091	0.00012
STRC	99.0	33.4	0.337	4.43 × 10^−7^
HGF	81.8	17.9	0.219	5.40 × 10^−7^
FSCN2	72.8	25.7	0.354	1.68 × 10^−5^
DACH1	60.4	12.6	0.210	0.00010
CD96	52.2	7.8	0.150	0.00019
PITX2	50.7	11.0	0.218	1.08 × 10^−7^
CDH12	47.9	15.3	0.320	3.48 × 10^−5^
ACVRL1	33.8	11.8	0.344	0.00568
CCL25	19.5	3.8	0.193	0.00095

## Data Availability

RNAseq data presented in this study are available as Digital Supplementary (Additional file S1. Array Data co vs sim.xlsx).

## Supplementary Materials

The following supporting information can be downloaded at: https://www.mdpi.com/article/10.3390/biomedicines11010029/s1, Figure S1. The amount of urokinase plasminogen activator (uPA) was determined in the cell culture supernatant of PCa cells by ELISA according to the manufacturer’s instructions. The uPA levels were normalized to cell number and expressed as percentage of control. Digital Supplementary, Additional file S1. Array Data co vs sim.xlsx.
