# Supplementary material for: Long-Term Treatment with Simvastatin Leads to Reduced Migration Capacity of Prostate Cancer Cells
Source: Biomedicines. 2022 Dec 22;11(1):29. doi: 10.3390/biomedicines11010029 (PMC9855777; doi:10.3390/biomedicines11010029)
Supplement: Supplementary file 1 [file biomedicines-11-00029-s001.zip › biomedicines-2096952- Suppl Figure S1 .pptx]

## Slide 1
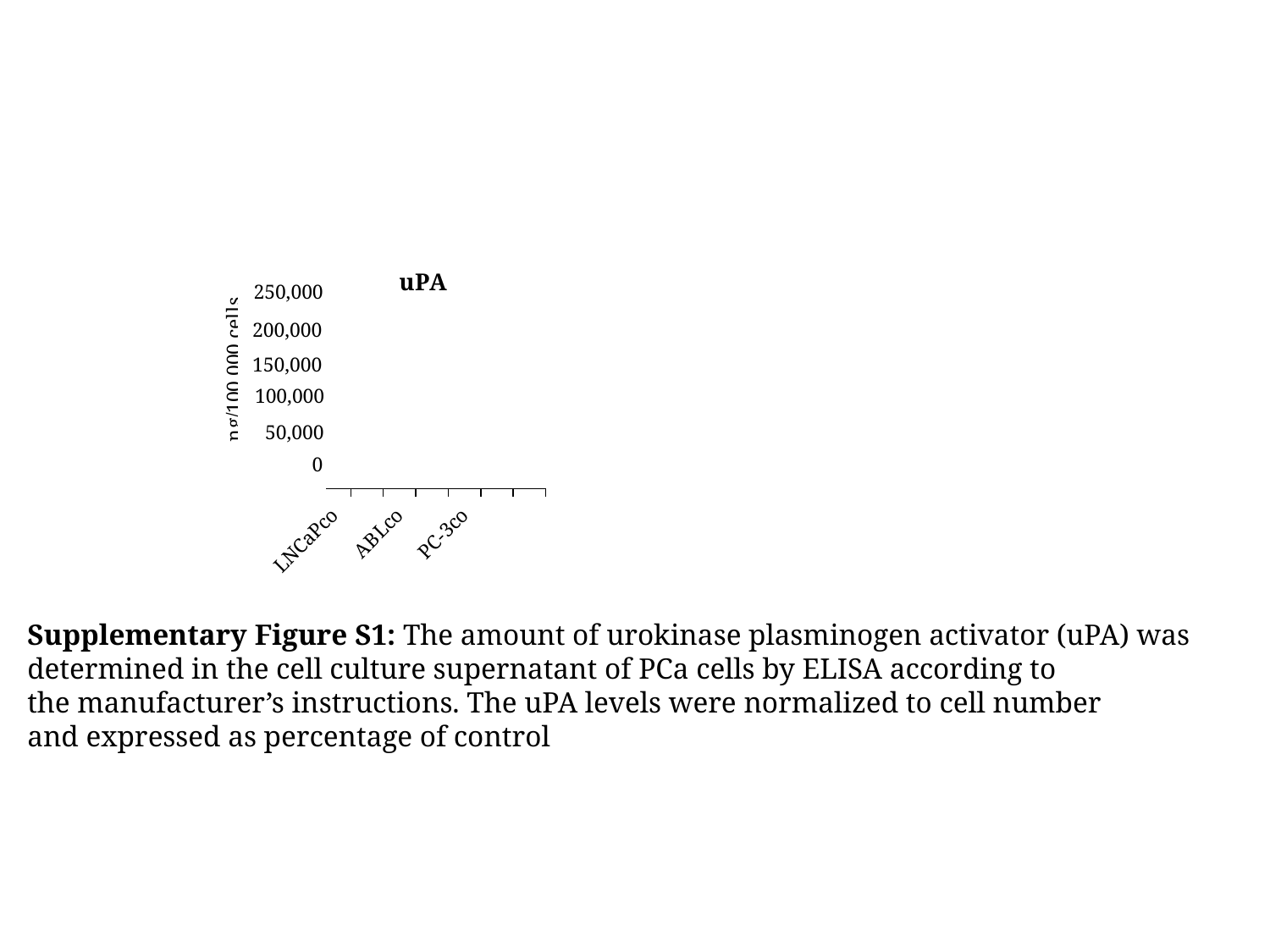

### Chart: uPA
| Category | Mean uPA(ng/100000 cells) |
|---|---|
| LNCaPco | 0.004 |
| LNCaPsim | 0.011 |
| ABLco | 0.066 |
| ABLsim | 0.009 |
| PC-3co | 137428.2126823085 |
| PC-3sim | 30198.311709651625 |250,000
200,000
150,000
100,000
50,000
0
Supplementary Figure S1: The amount of urokinase plasminogen activator (uPA) was
determined in the cell culture supernatant of PCa cells by ELISA according to
the manufacturer’s instructions. The uPA levels were normalized to cell number
and expressed as percentage of control
